# Tumor suppression and improvement in immune systems by specific activation of dopamine D1-receptor-expressing neurons in the nucleus accumbens

**DOI:** 10.1186/s13041-022-00902-1

**Published:** 2022-02-16

**Authors:** Daisuke Sato, Yusuke Hamada, Michiko Narita, Tomohisa Mori, Hiroyuki Tezuka, Yukari Suda, Kenichi Tanaka, Sara Yoshida, Hideki Tamura, Akihiro Yamanaka, Emiko Senba, Naoko Kuzumaki, Minoru Narita

**Affiliations:** 1 Department of Pharmacology, Hoshi University School of Pharmacy and Pharmaceutical Sciences, 2-4-41 Ebara, Shinagawaku, Tokyo 142-8501 Japan; 2 Division of Cancer Pathophysiology, National Cancer Center Research Institute (NCCRI), 5-1-1 Tsukiji, Chuo-ku, Tokyo, 104-0045 Japan; 3 Department of Molecular and Cellular Medicine, Institute of Medical Science, Tokyo Medical University, 6-7-1 Nishishinjuku, Shinjuku-ku, Tokyo, 160-0023 Japan; 4 Department of Cellular Function Analysis, Research Promotion and Support Headquarters, Fujita Health University, 1-98 Dengakugakubo, Kutsukake-cho, Toyoake, Aichi 470-1192 Japan; 5 Institute for Advanced Life Sciences, Hoshi University School of Pharmacy and Pharmaceutical Sciences, 2-4-41 Ebara, Shinagawa-ku, Tokyo, 142-0063 Japan; 6 Laboratory of Biofunctional Science, Hoshi University School of Pharmacy and Pharmaceutical Sciences, 2-4-41 Ebara, Shinagawa-ku, Tokyo, 142-0063 Japan; 7 Department of Neuroscience II, Research Institute of Environmental Medicine, Nagoya University, Furo-cho, Chikusa-ku, Nagoya, 464-8601 Japan; 8 Department of Physical Therapy, Osaka Yukioka College of Health Science, 1-1-41 Sojiji, Ibaraki, Osaka 567-0801 Japan; 9 Department of Rehabilitation Medicine, Wakayama Medical University, 811-1 Kimiidera, Wakayama, Wakayama 641-8509 Japan

## Abstract

Recent research has suggested that the mesolimbic dopamine network that mainly terminates in the nucleus accumbens may positively control the peripheral immune system. The activation of dopamine receptors in neurons in the nucleus accumbens by the release of endogenous dopamine is thus expected to contribute to efferent immune regulation. As in the stimulation of Gs-coupled dopamine D1-receptors or Gi-coupled D2-receptors by endogenous dopamine, we investigated whether specific stimulation of dopamine D1-receptor-expressing neurons or inhibition of dopamine D2-receptor-expressing neurons in the nucleus accumbens could produce anti-tumor effects and improve the immune system in transgenic mice using pharmacogenetic techniques. Repeated stimulation of D1-receptor-expressing neurons in either the medial shell, lateral shell or core regions of the nucleus accumbens significantly decreased tumor volume under a state of tumor transplantation, whereas repeated suppression of D2-receptor-expressing neurons in these areas had no effect on this event. The number of splenic CD8^+^ T cells was significantly increased following repeated stimulation of D1-receptor-expressing neurons in the nucleus accumbens of mice with tumor transplantation. Furthermore, this stimulation produced a significant reduction in the population of splenic CD8^+^ T cells that expressed immune checkpoint-related inhibitory receptors, PD-1, TIM-3 and LAG-3. These findings suggest that repeated stimulation of D1-receptor-expressing neurons (probably D1-receptor-expressing medium spiny neurons) in the nucleus accumbens suppressed tumor progression and improved the immune system by suppressing the exhaustion of splenic CD8^+^ T cells.

## Introduction

Dopamine is one of the most well-known neurotransmitters and its transmission in mesolimbic areas controls several physiological functions, such as the modulation of movement and reward [1]. Because of the multiple physiological functions of dopamine, dopaminergic dysfunction is considered to be involved in a variety of disease pathologies. For example, Parkinson’s disease has been well-recognized to be induced under a hypo-dopaminergic environment associated with a chronic state of neuroinflammation/neuroimmune abnormality [2, 3]. Interestingly, current evidence indicates that the brain’s reward projection that mainly terminates in the nucleus accumbens and modulates emotions may play a role in the efferent control of the immune system [4–6].

Cancer is a systemic disease that can weaken the immune system by affecting multiple functions of a variety of immune cells [7, 8]. A better understanding of the central and/or efferent immune modification of such systemic immune systems could provide a novel platform for cancer therapies.

The dopamine released from nerve terminals can activate dopamine receptors, including dopamine D1- and D2-receptors, in neurons, which tonically regulate motivation. A growing body of evidence suggests that approximately 95% of nucleus accumbens neurons are GABAergic medium spiny neurons (MSNs) [9]. There are two types of MSNs in the nucleus accumbens: dopamine D1-receptor-expressing MSNs (D1-MSNs) and dopamine D2-receptor-expressing MSNs (D2-MSNs) [10–12]. Little is known about the possible role of D1/D2-MSNs in the nucleus accumbens on the regulation of the immune system.

In the present study, we therefore investigated whether selective stimulation of D1-receptor-expressing neurons and selective suppression of D2-receptor-expressing neurons, both of which could be spontaneously regulated by endogenously released dopamine through the activation of dopamine D1-(Gs-coupling) and D2-(Gi-coupling) receptors, in the nucleus accumbens could produce anti-tumor effects in mice using pharmacogenetic techniques to directly manipulate neuronal activities. Furthermore, since emerging evidence suggests that dysfunctional immune cells, especially T cells, are involved in cancer pathology [13–15], the influence of the modulation of D1/D2-receptor-expressing neurons in the nucleus accumbens on T cell function in the spleen was also investigated under a state of tumor transplantation.

## Method

### Animals

Wild type mice (C57BL/6 N background) were either bred or purchased from CLEA Japan (Tokyo, Japan). D1-cre (C57BL/6-Drd1a < tm1(cre) > Phsh) (Cyagen Bioscience Inc., Santa Clara, CA, USA) and D2-cre (C57BL/6-Drd2 < tm1(cre) > Phsh) (Cyagen Bioscience Inc.) mice were used in the present study. D1-cre and D2-cre mice were created at Cyagen Bioscience using CRISPR Cas9 technology. All mice were housed at up to six mice per cage and kept in a temperature- and humidity-controlled room (24 ± 1 °C, 55 ± 5%, relative humidity) under a 12-h light–dark cycle (light on at 8:00 a.m. to 8:00 p.m.). Food and water were available ad libitum and behavioral experiments were performed in the light phase. All experiments were conducted in accordance with the Guide for Care and Use of Laboratory Animals of Hoshi University School of Pharmacy and Pharmaceutical Sciences.

### Drug

Clozapine *N*-oxide (CNO; Abcam plc., Cambridge, UK) was dissolved, and administered in a volume of 0.1 mL/10 g in saline (Otsuka Pharmaceutical Factory Inc., Tokushima, Japan).

### Virus

AAV9-hSyn-Flex-hM3Dq-mCherry (5 × 10^12^ copies/mL) and AAV9-hSyn-Flex-hM4Di-mCherry (4 × 10^12^ copies/mL) were used for the experiment of medial and lateral shell of the nucleus accumbens. AAV10-hSyn-Flex-hM3Dq-mCherry (9 × 10^12^ copies/mL) and AAV10-hSyn-Flex-hM4Di-mCherry (3 × 10^12^ copies/mL) were used for the experiment of core of the nucleus accumbens. All viruses were synthesized by us. These aliquots of virus were stored at -80 °C until use.

### Virus injection

Mice were placed in a stereotaxic apparatus (RWD Life Science, San Diego, CA, USA) under isoflurane (3%, inhalation) anesthesia, and the skull was exposed. A small hole was then made using a dental drill. Virus was bilaterally injected into the nucleus accumbens core (from the bregma: AP + 1.4 mm, ML ± 1.5 mm, DV -3.6 mm from the brain surface at an angle of 10°: 1.0 μL was applied to each side using a Hamilton syringe), the lateral shell (from the bregma: AP + 1.0 mm, ML ± 1.8 mm, DV -4.9 mm from the skull at an angle of 0°: 300 nL was applied to each side using a Nanoject III (Drummond Scientific Company, Broomall, PA, USA)) and the medial shell (from the bregma: AP + 1.5 mm, ML ± 0.5 mm, DV − 4.7 mm from the skull at an angle of 0°: 300 nL was applied to each side using a Nanoject III (Drummond Scientific Company)).

### Electrophysiological validation of hM3Dq and hM4Di activation

Coronal brain slices (250 μm) containing the nucleus accumbens were prepared with a vibratome (VT-1200S, Leica Biosystems, Wetzlar, Germany), using ice-cold cutting solution containing (in mM) 222.1 sucrose, 2.5 KCl, 1 CaCl_2_, 7 MgSO_4_, 1.4 NaH_2_PO_4_, 27 NaHCO_3_, and 0.5 ascorbic acid (oxygenated with 95% O_2_/5% CO_2_). Slices were recovered for at least 1 h at room temperature in oxygenated artificial cerebrospinal fluid containing (in mM) 128 NaCl, 3 KCl, 2 CaCl_2_, 2 MgCl_2_, 1.25 NaH_2_PO_4_, 24 NaHCO_3_, and 10 glucose. The fluorescence of the cells in the nucleus accumbens was detected by an upright fluorescence microscope (ECLIPSE FN1; Nikon, Tokyo, Japan) using a 40 × water-immersion objective and a sCMOS camera (Zyla 5.5 sCMOS; Andor Technology, Belfast UK). Whole-cell patch-clamp recording was made from D1- and D2-receptor-expressing neurons with hM3Dq-mCherry and hM4Di-mCherry (3 slices per animal), respectively, with a Multiclamp 700B Amplifier (Molecular Devices, Sunnyvale, CA, USA). The recording electrodes are borosilicate glass pipettes (4–6 MΩ) and filled with the following solution: (in mM): 120 potassium gluconate, 10 KCl, 10 HEPES, 10 phosphocreatine-Na_2_, 4 Mg_2_ATP, 0.3 Na_3_GTP, 0.2 EGTA, and 0.04 Alexa Fluor 488 hydrazide dye (pH 7.3 with KOH). Data were stored with pCLAMP 10 software (Molecular Devices). CNO (3 µM) was added into the superfusion medium to activate the hM3Dq and hM4Di receptor. After recording, slices were fixed in 4% paraformaldehyde, and then mounted on glass slides using Prolong Diamond Antifade Reagent (Thermo Fisher Scientific, Inc. Waltham, MA, USA). Fluorescent 2D projection images were obtained from a series of z-stack images (each, 3.6 μm thick at 0.6 μm intervals) using a confocal laser scanning microscope (FV3000; Olympus, Tokyo, Japan) equipped with a 40 × /0.96 NA objective at 488 nm for Alexa Fluor 488 and 561 nm for mCherry.

### Graft tumor growth assay

Mice were anesthetized by 3% isoflurane. Lewis lung carcinoma (LLC) cells were resuspended in a mixture of extracellular matrix (ECM) gel (Sigma-Aldrich, St. Louis, MO, USA) and hank’s Balanced Salt solution (HBSS; Thermo Fisher Scientific, Inc.) (ratio 3:1). The cell suspension (2 × 10^6^ cells/0.5 mL/mouse) was inoculated subcutaneously into the right lower back of mice. Tumor size was measured using calipers, whereas tumor volume was calculated as (L × W^2^)/2, where L = length and W = width. Tumor size was measured every other day for 14 days. To investigate the effects of the stimulation of D1-receptor-expressing neurons by hM3Dq or inhibition of D2-receptor-expressing neurons by hM4Di in the nucleus accumbens on tumor growth, CNO (3 mg/kg, i.p., t.i.d.) was administered to mice.

### Flow cytometry

Fifteen days after the transplantation of LLC cells, the spleen was isolated under isoflurane anesthesia (3%, inhalation), and then homogenized with phosphate-buffered saline (PBS) by pipetting. To remove cell aggregates, the homogenized suspension was passed through a 100-μm cell strainer and a nylon mesh. Subsequently, ammonium chloride was applied to a single-cell suspension for hemolysis, and the cell suspension was fractionated at 6 × 10^6^ cells/tube. For blocking, cells were treated with an anti-CD16/32 antibody (BD Biosciences, Inc.). Cells were stained with each antibodies; T cells (CD45.2-APC/Cy7, CD4-FITC, CD8-PE), NK and NKT cells (CD45.2-APC/Cy7, CD3ε-PE, CD49b-APC), neutrophil (CD45.2-APC/Cy7, Ly6G-PE/Cy7, CD11b-FITC), macrophages (CD45.2-APC/Cy7, F4/80-PE) and exhausted CD8^+^ T cell (CD45.2-APC/Cy7, CD3-FITC, CD8-PE, CD279 (PD-1)-APC, CD366 (TIM-3)-PE/Cy7 or CD223 (LAG-3)-PE/Cy7). All antibodies were purchased from Bio Legend (San Diego, CA, USA). Dead cells were stained by propidium iodide (PI; Sigma-Aldrich). Immune cells were sorted using a BD FACS Aria™ II Cell Sorter (BD Biosciences) and then analyzed.

### Statistical analysis

The data are presented as the mean ± S.E.M. All statistical analyses were performed with GraphPad Prism 8.0 (GraphPad Software, San Diego, CA, USA). The statistical significance of differences between the groups was assessed by the Mann–Whitney test or a two-way repeated measures analysis of variance followed by the Bonferroni multiple comparisons test. Correlation analysis was performed by Pearson's correlation coefficient test. A p value of < 0.05 was considered to reflect significance.

## Results

### Activation of D1-receptor-expressing neurons suppresses tumor growth

**Fig. 1:**
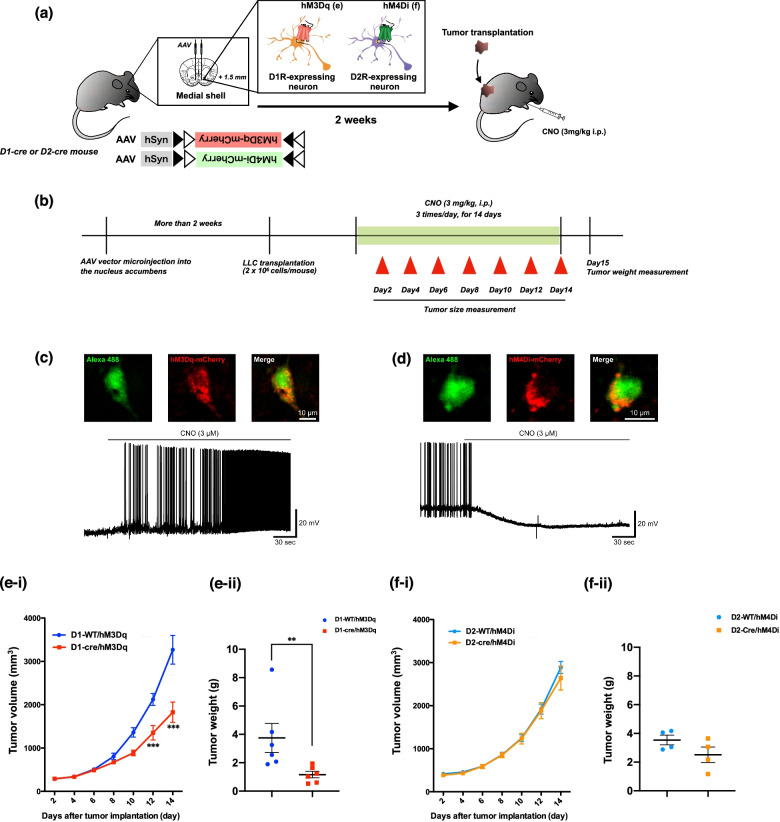
Effect of pharmacogenetic activation of D1-receptor-expressing neurons in the medial shell of the nucleus accumbens on tumor growth. **a** Schematic diagram showing experimental design. The D1-WT or D1-Cre mice were microinjected with hM3Dq into the medial shell of the nucleus accumbens. Two weeks after the microinjection, mice were transplanted by LLC cells and administered repeatedly by CNO (3 mg/kg, i.p., t.i.d.). **b** Experimental timeline. **c**, **d** Confocal images of Alexa 488 hydrazide-stained D1-hM3Dq-mCherry positive neurons (**c**) or D2-hM4Di-mCherry positive neurons **(d**). Scale Bar = 10 µm. Current-clamp traces of D1-hM3Dq-positive (**c**) and D2-hM4Di-positive (**d**) neurons by CNO treatment in the nucleus accumbens. **e** Suppression of the increases in tumor volume (**e-i**, Two-way repeated measures ANOVA with post-hoc Bonferroni test, ^***^p < 0.001 vs D1-WT/hM3Dq) and tumor weight (**e-ii**, Mann–Whitney test, ^**^p < 0.01 vs D1-WT/hM3Dq) by concomitant stimulation of D1-receptor-expressing neurons through activation of hM3Dq in the medial shell of the nucleus accumbens of D1-Cre mice following repeated administration of CNO (3 mg/kg, i.p., t.i.d.) in comparison to those of D1-WT mice. **f** Effects of concomitant inhibition of D2-MSNs through stimulation of hM4Di in the medial shell of the nucleus accumbens of D2-cre mice by repeated administration of CNO (3 mg/kg, i.p., t.i.d.) on tumor volume (**f-i**) and tumor weight (**f-ii**) in comparison to those in D2-WT mice. Each point represents the mean ± S.E.M. of 4–6 animals

**Fig. 2:**
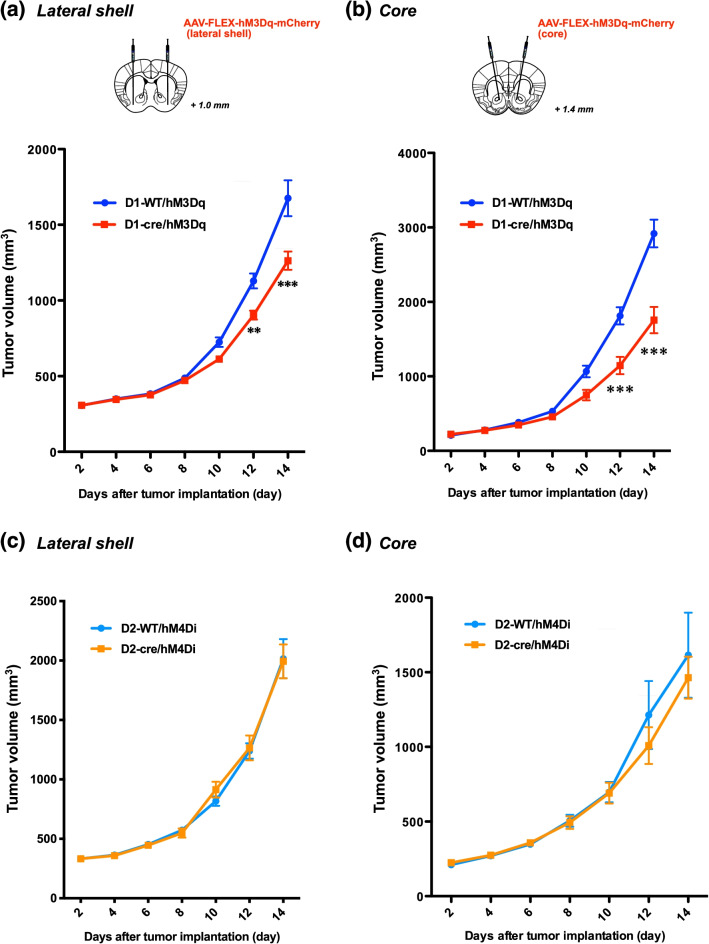
Effect of pharmacogenetic activation of D1-receptor-expressing neurons in the lateral shell or core of the nucleus accumbens on tumor growth. **a**, **b** Suppression on the tumor volume by concomitant stimulation of D1-receptor-expressing neurons through activation of hM3Dq in the lateral shell (**a**) or core (**b**) of the nucleus accumbens of D1-Cre mice by repeated administration of CNO (3 mg/kg, i.p., t.i.d.) in comparison of those of D1-WT mice (Two-way repeated measures ANOVA with post-hoc Bonferroni test, ^**^p < 0.01, ^***^p < 0.001 vs. D1-WT/hM3Dq). **c**, **d** Effects of concomitant inhibition of D2-receptor-expressing neurons through stimulation of hM4Di in the lateral shell (**c**) or core (**d**) of the nucleus accumbens of D2-cre mice by repeated administration of CNO (3 mg/kg, i.p., t.i.d.) on tumor volume in comparison to those in D2-WT mice. Each point represents the mean ± S.E.M. of 4–11 animals

Following the Cre-loxP system, D1-cre or D2-cre mice were injected with a Cre-dependent AAV carrying a construct of hM3Dq or hM4Di into the medial shell of the nucleus accumbens, respectively (Fig. 1a, b). Whole-cell patch-clamp recording showed that D1-cre/hM3Dq- or D2-cre/hM4Di-positive neurons in the nucleus accumbens clearly responded to treatment with CNO (Fig. 1c, d). To investigate whether activation of D1-receptor-expressing neurons or suppression of D2-receptor-expressing neurons in the medial shell of the nucleus accumbens could produce an anti-tumor effect, changes in tumor size were measured after LLC cell transplantation. As a result, when tumor volume was gradually increased in a time-dependent manner after the transplantation of LLC cells, activation of D1-receptor-expressing neurons in the medial shell of the nucleus accumbens by the Gq-designer receptors exclusively activated by designer drugs (DREADD) system significantly decreased tumor volume compared to that in D1-WT/hM3Dq mice (Fig. 1e-i, Two-way repeated measures ANOVA with post-hoc Bonferroni test, ^***^p < 0.001 vs D1-WT/hM3Dq). In addition, stimulation of D1-receptor-expressing neurons in the medial shell of the nucleus accumbens was associated with dramatic decreases in tumor weight (Fig. 1e-ii, Mann–Whitney test, ^**^p < 0.01 vs D1-WT/hM3Dq). On the other hand, specific suppression of D2-receptor-expressing neurons had no effect on tumor volume or tumor weight (Fig. 1f-i, f-ii). We examined the anti-tumor effect of D1-receptor-expressing neurons in other regions of the nucleus accumbens, and found that activation of D1-receptor-expressing neurons in the lateral shell and core, as well as medial shell, of the nucleus accumbens significantly decreased tumor volume (Fig. 2a, b, Two-way repeated measures ANOVA with post-hoc Bonferroni test, ^**^p < 0.01, ^***^p < 0.001 vs D1-WT/hM3Dq). However, specific suppression of D2-receptor-expressing neurons in the lateral shell or core of the nucleus accumbens had no effect on tumor volume (Fig. 2c, d).

### Activation of D1-receptor-expressing neurons decreases the exhaustion of immune cells

**Fig. 3:**
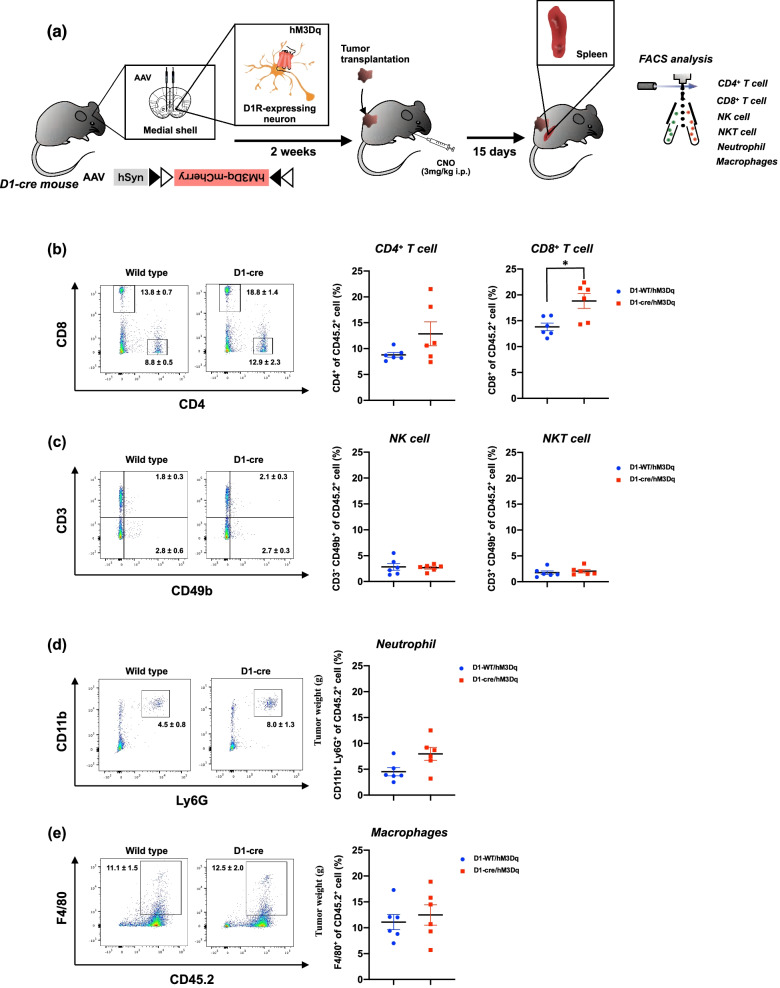
Effect of pharmacogenetic activation of D1-receptor-expressing neurons in the medial shell of the nucleus accumbens on the systemic immune response. **a** Schematic diagram of the experimental design. The D1-WT or D1-Cre mice were microinjected with hM3Dq into the medial shell of the nucleus accumbens. Two weeks after the microinjection, mice were transplanted by LLC cells and administered repeatedly by CNO (3 mg/kg, i.p., t.i.d.). Fifteen days after LLC transplantation and CNO administration, the spleen was harvested from these mice and homogenized to make a single-cell suspension followed by flow cytometry to analyze the immune cells in the spleen. **b**–**e** Representative flow cytometric plots (left panel) and quantitative evaluation of the number (right panel) of CD4^+^ T and CD8^+^ T cells (**b**), NK and NKT cells (**c**), neutrophil (**d**) and macrophages (**e**) derived from the spleen of D1-WT/hM3Dq and D1-Cre/hM3Dq mice bearing LLC after repeated administration of CNO. Each data point represents the mean ± S.E.M. of 6 samples (Mann–Whitney test, ^*^p < 0.05 vs D1-WT/hM3Dq)

**Fig. 4:**
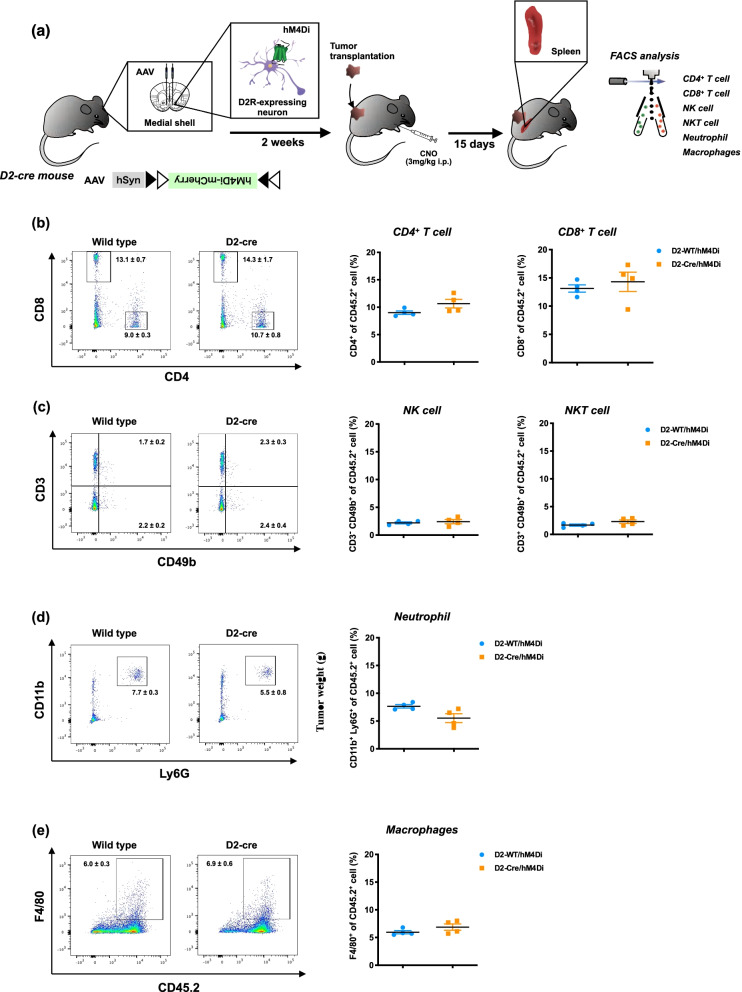
Effect of pharmacogenetic suppression of D2-receptor-expressing neurons in the medial shell of the nucleus accumbens on the systemic immune response. **a** Schematic diagram showing experimental design. The D2-WT or D2-Cre mice were microinjected with hM4Di into the medial shell of the nucleus accumbens. Two weeks after the microinjection, mice were transplanted by LLC cells and administered repeatedly by CNO (3 mg/kg, i.p., t.i.d.). Fifteen days after LLC transplantation and CNO administration, the spleen was harvested from these mice and homogenized to make a single-cell suspension followed by flow cytometry to analyze the immune cells in the spleen. **b**–**e** Representative flow cytometric plots (left panel) and quantitative evaluation of the number (right panel) of CD4^+^ T and CD8^+^ T cells (**b**), NK and NKT cells (**c**), neutrophil (**d**) and macrophages (**e**) derived from the spleen of D2-WT/hM4Di and D2-Cre/hM4Di mice bearing LLC after repeated administration of CNO, which were microinjected with hM4Di. Each data point represents the mean ± S.E.M. of 4 samples

We next investigated possible changes in the number of leucocytes from the spleen of D1-cre/hM3Dq mice following specific stimulation of D1-receptor-expressing neurons in the medial shell region of the nucleus accumbens using FACS (Fig. 3a). Repeated stimulation of D1-receptor-expressing neurons in the medial shell of the nucleus accumbens failed to change the number of CD4^+^ T cells (Fig. 3b), NK cells (Fig. 3c), NKT cells (Fig. 3c), neutrophil (Fig. 3d) or macrophages (Fig. 3e) in the spleen of mice compared to those in D1-WT/hM3Dq mice at 15 days after LLC transplantation. A significant increase in the number of CD8^+^ T cells in the spleen was observed following repeated stimulation of D1-receptor-expressing neurons in the medial shell of the nucleus accumbens of mice with LLC transplantation compared to that in D1-WT/hM3Dq mice (Fig. 3b, Mann–Whitney test, ^*^p < 0.05 vs D1-WT/hM3Dq). We also analyzed the immune cells in the spleen when D2-receptor-expressing neurons in the medial shell of the nucleus accumbens were suppressed by hM4Di (Fig. 4a). Repeated suppression of D2-receptor-expressing neurons in the medial shell of the nucleus accumbens failed to change the number of CD4^+^ T cells (Fig. 4b), CD8^+^ T cells (Fig. 4b), NK cells (Fig. 4c), NKT cells (Fig. 4c), neutrophil (Fig. 4d) or macrophages (Fig. 4e) in the spleen of mice compared to those in D2-WT/hM4Di mice at 15 days after LLC transplantation.

**Fig. 5:**
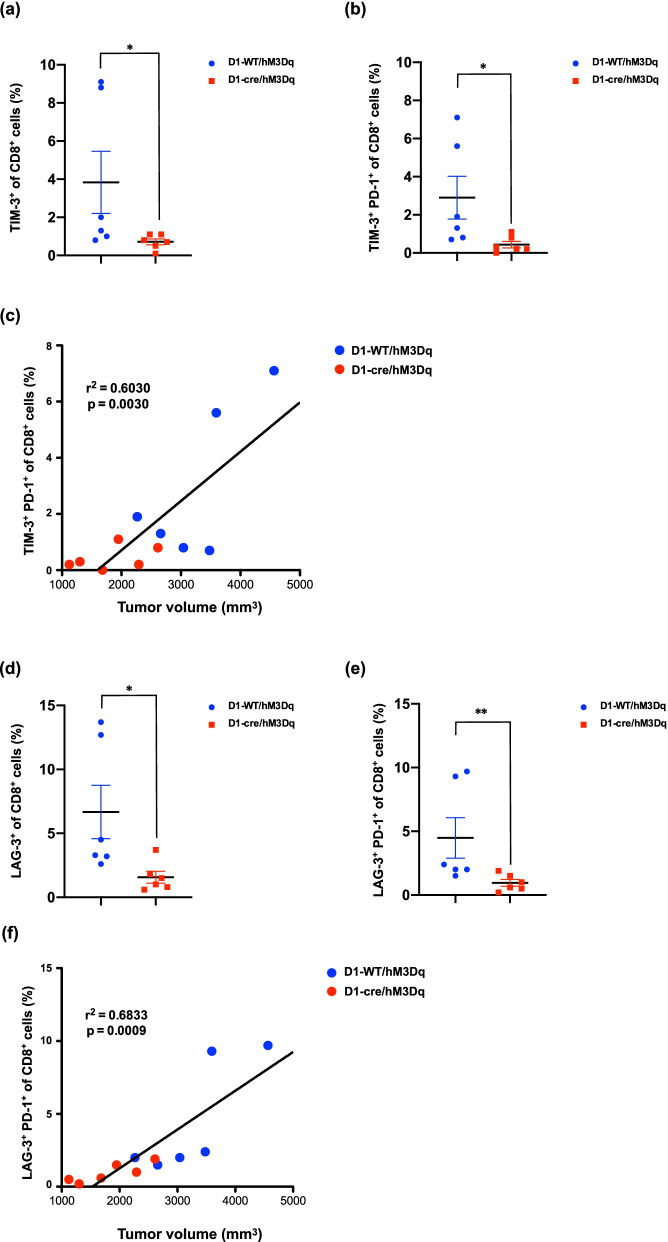
Effect of pharmacogenetic activation of D1-receptor-expressing neurons in the medial shell of the nucleus accumbens on the state of exhaustion of CD8^+^ T cells. **a**, **b** Effects of activation of D1-receptor-expressing neurons by hM3Dq in the medial shell of the nucleus accumbens of D1-cre mice with hM3Dq by repeated administration of CNO on TIM-3^+^ (**a**) and TIM-3^+^ PD-1^+^ (**b**) levels of CD8^+^ T cells in the spleen 15 days after LLC transplantation (Mann–Whitney test, *p < 0.05 vs. D1-WT/hM3Dq). **c** Relationship between the tumor volume and percentage of TIM-3^+^ PD-1^+^ in CD8^+^ T cells from the spleen of D1-WT and D1-Cre mice with hM3Dq, which had been implanted with LLC, after activation of D1-receptor-expressing neurons in the medial shell of the nucleus accumbens (Pearson's correlation coefficient test, r^2^ = 0.6030, p = 0.0030). **d**, **e** Effects of activation of D1-receptor-expressing neurons in the medial shell of the nucleus accumbens of D1-cre mice with hM3Dq by repeated administration of CNO on LAG-3^+^ (**d**) and LAG-3^+^ PD-1^+^ (**e**) levels of CD8^+^ T cells in the spleen 15 days after LLC transplantation (Mann–Whitney test, *p < 0.05, **p < 0.01 vs. D1-WT/hM3Dq). (**f**) Relationship between tumor volume and the percentage of LAG-3^+^ PD-1^+^ in CD8^+^ T cells from the spleen of D1-WT and D1-Cre mice with hM3Dq, which had been implanted with LLC, after activation of D1-receptor-expressing neurons in the medial shell of the nucleus accumbens by repeated administration of CNO (Pearson's correlation coefficient test, r^2^ = 0.6833, p = 0.0009). Each point represents the mean ± S.E.M. of 6 samples

To further assess the effect of D1-receptor-expressing neurons stimulation in the medial shell of the nucleus accumbens on the function of CD8^+^ T cells, we next investigated the possible changes in the population of CD8^+^ T cells with the expression of immune checkpoint-related inhibitory receptors, such as programmed cell death protein-1 (PD-1), T cell immunoglobulin and mucin domain-containing protein 3 (TIM-3) and Lymphocyte-activation-gene 3 (LAG-3), in the spleen of mice with tumor transplantation. Under these conditions, the number of either TIM-3^+^ CD8^+^ T cells (Fig. 5a, Mann–Whitney test, ^*^p < 0.05 vs D1-WT/hM3Dq) or LAG-3^+^ CD8^+^ T cells (Fig. 5d, Mann–Whitney test, ^*^p < 0.05 vs D1-WT/hM3Dq) in the spleen was significantly decreased by repeated stimulation of D1-receptor-expressing neurons in the medial shell of the nucleus accumbens of tumor-bearing mice. In addition, repeated stimulation of D1-receptor-expressing neurons in this region of tumor-bearing mice produced further decrease in the number of either TIM-3^+^ PD-1^+^ CD8^+^ T cells (Fig. 5b, Mann–Whitney test, ^*^p < 0.05 vs D1-WT/hM3Dq) or LAG-3^+^ PD-1^+^ CD8^+^ T cells (Fig. 5e, Mann–Whitney test, ^**^p < 0.01 vs D1-WT/hM3Dq) in the spleen. The number of either TIM-3^+^ PD-1^+^ CD8^+^ T cells or LAG-3^+^ PD-1^+^ CD8^+^ T cells was significantly correlated with tumor volume following repeated stimulation of D1-receptor-expressing neurons in the medial shell of the nucleus accumbens in mice transplanted with LLC (Fig. 5c, f, Pearson's correlation coefficient test).

## Discussion

It has been empirically considered that autonomic and emotional regulation may strongly affect the immune system [16–18]. While it is clear that the receptors and synthetic pathways of dopamine are present in leukocytes [19–23], alterations in the activity of the central dopaminergic system, which play an important role in the development of neuropsychiatric disorders such as Parkinson's disease and schizophrenia, may not only directly trigger the emotion-immune-associated efferent system, but also create a "vicious efferent-afferent cycle" in which the concomitant development of immune abnormalities leads to exacerbation of these symptoms. For example, a large cohort study found a lower incidence of cancer in patients with schizophrenia, the predominant cause of which is central dopaminergic overactivity, compared with that in the general population [24]. The cytokine balance in plasma of untreated schizophrenic patients has been reported to shift to Th1 cells compared to those in healthy individuals and patients receiving antipsychotics [25]. However, the relationship between central dopamine-related signaling and the peripheral immune system has long been a matter of debate on the assumption that most may be due to dopamine released from nodal nerve terminals, and the effects of central dopamine responses on peripheral immune systems have not been elucidated. Recently, it has been reported that specific stimulation of dopaminergic neurons in the ventral tegmental area has a beneficial effect on the peripheral immune system and has a strong bactericidal effect against bacterial infection [5, 6], indicating that activation of mesolimbic dopamine neurons enhances immune function. Although this report has attracted attention to the link between the central dopaminergic network and the immune network, little is still known about the central dopaminergic-peripheral immune linkage, and there are no systematic reports on the effects of downstream signals in the central dopaminergic network on tumor growth. Here, we found that repeated activation of D1-receptor-expressing neurons in the shell and core regions of the nucleus accumbens significantly decreased tumor volume as well as tumor weight compared to those in a control group. These findings provide evidence that activated D1-receptor-expressing neurons in the nucleus accumbens may lead to tumor suppression.

To clarify the mechanisms of the anti-tumor effects of the stimulation of D1-receptor-expressing neurons in the nucleus accumbens, we next evaluated the possible changes in the number of leucocytes from the spleen of D1-Cre/hM3Dq mice by specific stimulation of the D1-receptor-expressing neurons in the medial shell region of the nucleus accumbens, and found a significant increase in the number of CD8^+^ T cells in the spleen following repeated stimulation of D1-receptor-expressing neurons in the medial shell of the nucleus accumbens. It is well known that common bone marrow-derived progenitor cells differentiate into three types of T cells with different functions in the thymus [26, 27]. Among these, T cells that express αβ T cell receptors differentiate into CD4 or CD8 single-positive cells. They then leave the thymus and enter the systemic circulation as mature naive CD4^+^ T cells or CD8^+^ T cells. These naive T cells circulate in the blood and lymph system and become activated and differentiate into effector T cells in secondary lymphoid tissues such as lymph nodes, spleen, and mucosa-associated lymphoid tissue when they encounter specific antigens presented by dendritic cells. Especially, effector CD8^+^ T cells, called cytotoxic T cells, play an important role in immune defense against tumors and intracellular pathogens such as viruses and bacteria. Considering the immune system, we hypothesize here that concomitant activation of D1-receptor-expressing neurons in the nucleus accumbens may suppress tumor progression, at least in part, via an increase in the population of effector CD8^+^ T cells.

Unlike D1-receptor-expressing neurons, we found that repeated inhibition of D2-receptor-expressing neurons in the shell and core regions of the nucleus accumbens, which could parallel the state of the stimulation of D2-receptors by released dopamine in D2-receptor-expressing neurons because of essential D2-Gi coupling, failed to decrease tumor volume or change the number of effector T cells compared to those in a control group. In a preliminary experiment, we found that concomitant stimulation of D2-receptor-expressing neurons in the medial shell of the nucleus accumbens had no effect on tumor growth (data not shown). Taken together, these findings indicate that D2-receptor-expressing neurons in the nucleus accumbens may not be involved in the efferent modulation of tumor suppression and progression. Although further investigation should be required, these differences in the modulation of D1- and D2-receptor-expressing neurons may result from the difference in efferent projections between D1-MSNs and D2-MSNs [28–30]. On the other hand, we still cannot deny the possibility that different experimental conditions, such as a different frequency or time period of the suppression of D2-receptor-expressing neurons, may cause the different results.

We have no doubt that CD8^+^ T cells play an important role in the protective response to intracellular pathogens and tumors. However, in the presence of chronic infections and tumors, CD8^+^ T cells are constantly exposed to antigens and inflammatory signaling molecules, causing functional deterioration called “cell exhaustion”, which results in chronic infection and tumor growth [31, 32]. These exhausted T cells express high levels of multiple inhibitory receptors, such as PD-1, TIM-3 and LAG-3, leading to a loss of the T cell's own proliferative capacity and a decrease in effector function, cytokine production and killing function [33]. In addition, exhausted CD8^+^ T cells can be divided into pre-exhausted T cells and exhausted T cells. Although the former express PD-1, but are capable of killing tumor cells, the latter express high levels of PD-1, TIM-3 and LAG-3, and have a very low capacity to kill tumor cells. Another key finding of the present study was that the number of either TIM-3^+^ CD8^+^ T cells or LAG-3^+^ CD8^+^ T cells in the spleen was significantly decreased by the activation of D1-receptor-expressing neurons in the medial shell of the nucleus accumbens of tumor-bearing mice. Furthermore, these CD8^+^ T cells also showed a low expression of PD-1. Notably, the number of either PD-1^+^ TIM-3^+^ CD8^+^ T cells or PD-1^+^ LAG-3^+^ CD8^+^ T cells was significantly correlated with tumor volume following repeated stimulation of D1-receptor-expressing neurons in the nucleus accumbens under the condition of tumor transplantation. These findings support the idea that repeated stimulation of D1-receptor-expressing neurons in the nucleus accumbens may suppress the exhaustion of splenic CD8^+^ T cells under tumor transplantation. Since effector CD8^+^ T cells in the spleen could enhance the systemic immune defense against intracellular pathogens and tumors through the release of cytotoxic substances and exhausted CD8^+^ T cells may induce immune deterioration, we propose here that both an increase in the population of splenic CD8^+^ T cells and suppression of the exhaustion of splenic CD8^+^ T cells by repeated stimulation of D1-receptor-expressing neurons in the nucleus accumbens may contribute to tumor suppression through the facilitation of systemic immune systems.

We now raise the critical question of how the down-signaling of D1-receptor-expressing neurons after concomitant stimulation of D1-receptor-expressing neurons in the nucleus accumbens is conveyed to the peripheral immune system. Although we cannot clearly address this question at present, we suspect the possible involvement of symphonic modulation of endocrine mechanisms and sympathetic nervous system, but not the hypothalamus–pituitary–adrenal axis, in this event, which may affect all lymphoid organs and eventually modulate peripheral immune responses. In fact, we found a dynamic reduction in the increased levels of monocyte chemotactic protein-1 (MCP-1), macrophage inflammatory protein-2 (MIP-2), keratinocyte-derived chemokine (KC) and monokine induced by gamma interferon (MIG) in plasma following repeated stimulation of D1-receptor-expressing neurons in the nucleus accumbens, whereas the increased level of released cortisol in plasma of tumor-bearing mice was not affected by concomitant stimulation of D1-receptor-expressing neurons in the nucleus accumbens (data not shown).

Despite unknown down-signaling mechanisms, we demonstrated that activation of D1-receptor-expressing neurons in the nucleus accumbens region after the concomitant release of endogenous dopamine may suppress tumor progression through the efferent facilitation of the systemic immune system. Therefore, it is possible that anti-Parkinson agents that prefer to stimulate D1-receptors would be expected to show anti-tumor effects. As well as anti-Parkinson agents, exercise therapy may also be a promising tool, because voluntary exercise has been demonstrated to activate VTA dopamine neurons in mice [34, 35]. In fact, exercise could suppress tumor growth in mouse tumor models through the direct regulation of NK cell mobilization and trafficking [36]. Taken together, we hypothesize that exercise-induced activation of VTA dopamine neurons, which in turn stimulates D1-receptors in the nucleus accumbens, may be partly associated with its anti-tumor activity.

In conclusion, we found for the first time that repeated stimulation of D1-receptor-expressing neurons (probably D1-MSNs) in the nucleus accumbens region suppressed tumor progression and improved the immune system due to the increased population of CD8^+^ T cells in the spleen and the reduction in the population of exhausted splenic CD8^+^ T cells. These findings support the idea that activation of D1-MSNs in the nucleus accumbens region, which could be induced by the release of endogenous dopamine, may be a valuable and useful approach for cancer therapy.

## Data Availability

All of the data generated and analyzed in this study are included in this published article.

## Abbreviations

CNO: Clozapine *N*-oxide
D1-MSNs: Dopamine D1 receptor-expressing medium spiny neurons
D2-MSNs: Dopamine D2 receptor-expressing medium spiny neurons
DREADD: Designer receptors exclusively activated by designer drugs
FACS: Fluorescence-activated cell sorting
hM3Dq: Human muscarinic receptor 3 DREADD subtype
hM4Di: Human muscarinic receptor 4 DREADD subtype
LAG-3: Lymphocyte-activation gene 3
LLC: Lewis lung carcinoma
MSNs: Medium spiny neurons
PD-1: Programmed cell death protein 1
TIM-3: T cell immunoglobulin and mucin domain-containing protein 3
